# Fu’s subcutaneous needling as an adjunctive therapy for diaphragmatic dysfunction in a critically ill patient with severe neurologic disability: A case report

**DOI:** 10.1097/MD.0000000000035550

**Published:** 2023-11-03

**Authors:** Hu Li, Cong Cong Yang, Tianyu Bai, Jian Sun, Zhonghua Fu, Li-Wei Chou

**Affiliations:** a Shandong Provincial Third Hospital, Shandong University, Jinan, China; b Shandong University of Traditional Chinese Medicine, Jinan, China, ^c^ Clinical Medical College of Acupuncture and Moxibustion and Rehabilitation, Guangzhou University of Chinese Medicine, Guangzhou, China; d Second Clinical Medical College, Guangzhou University of Chinese Medicine, Guangzhou, China; e Institute of Fu’s Subcutaneous Needling, Beijing University of Chinese Medicine, Beijing, China; f Department of Physical Medicine and Rehabilitation, China Medical University Hospital, Taichung, Taiwan; g Department of Physical Therapy and Graduate Institute of Rehabilitation Science, China Medical University, Taichung, Taiwan; h Department of physical Medicine and Rehabilitation, Asia University Hospital, Asia University, Taichung, Taiwan.

**Keywords:** acupuncture, case report, diaphragmatic dysfunction, dry needling, Fu’s subcutaneous needling, respiratory rehabilitation

## Abstract

**Rationale:**

Diaphragmatic dysfunction is prevalent among intensive care unit patients. The use of Fu’s subcutaneous needling (FSN) for respiratory problems is a new issue and few study has been conducted so far.

**Patient concerns:**

Despite conventional treatments, the patient continued using noninvasive ventilation after discharge from the intensive care unit due to diaphragmatic dysfunction.

**Diagnosis:**

Diaphragmatic dysfunction.

**Interventions:**

After the myofascial trigger points were confirmed in the neck, chest, and abdomen area, FSN therapy was performed using disposable FSN needles. FSN needles were penetrated into the subcutaneous layer.

**Outcomes:**

The patient dyspnea and tachypnea improved, and noninvasive ventilation time dropped significantly. The patient was successfully weaned from the ventilator after 3 sessions of FSN therapy, and there was an increase in diaphragmatic excursion and tidal fraction of the diaphragm via the ultrasound imaging. We found no evidence of relapse 12 months after treatment.

**Lessons:**

FSN therapy has potential as an alternative strategy for patients with diaphragmatic dysfunction and severe neurologic disabilities who do not respond well to conventional therapies, but further research is still required to establish the effects of FSN on diaphragmatic function.

## 1. Introduction

Diaphragmatic dysfunction, which is prevalent among intensive care unit (ICU) patients, has not only been associated with prolonged ventilation, challenges in weaning, and increased mortality, but also significantly predicts ICU readmission after discharge.^[1,2]^ Studies have shown that patients with respiratory failure at ICU discharge often suffer from diaphragmatic dysfunction, which subsequently leads to the development of chronic critical illness and negatively impacts patients and their families.^[3]^

Enhancing clinical awareness of the effects of critical illness on respiratory muscles and diaphragmatic function, coupled with prioritizing preventative measures are essential to improving patient outcomes. Studies have shown that respiratory muscle rehabilitation can improve diaphragmatic strength and aid in weaning critically ill patients from ventilation.^[4,5]^ However, a myriad of barriers, such as the requirement for the patient to be alert and cooperative, compromise the implementation of respiratory muscle rehabilitation in critically ill patients.^[6]^ This precludes respiratory muscle rehabilitation for patients with significant neurological illness or cognitive impairment.

Fu’s subcutaneous needling (FSN), originating from Chinese acupuncture and dry needling has emerged as a popular treatment technique, but studies are largely limited to the treatment of myofascial trigger point (MTrP)-induced musculoskeletal disorders.^[7–9]^ From the perspective of FSN, the limb muscles can impact neighboring tissues and organs, including the respiratory system smooth muscles, leading to clinical manifestations such as chronic cough, asthma, shortness of breath, and breathing difficulties.^[10]^ The use of FSN for respiratory problems is a new issue and few studies have been conducted so far. Therefore, we report the first case report of FSN as an adjunctive therapy for treating diaphragmatic dysfunction in a critically ill patient with a severe neurologic disability.

## 2. Case presentation

An 18-year-old male patient with a history of hypoxic-ischemic brain injury, resulting in severe motor and language dysfunction as well as mild cognitive impairment, was admitted to a rehabilitation ward. He subsequently developed a high fever, recurrent cough, and dyspnea, which later worsened. He was transferred to the ICU for 16 days due to breathing problems. Upon stabilization, the patient was returned to the rehabilitation ward with persistent symptoms of dyspnea and tachypnea (30–40 breaths per minute). This necessitated continuous post-extubating noninvasive ventilation, which limited the ability to receive aggressive rehabilitation therapy. He was subjected to 24 hours of noninvasive ventilation for the first 4 days, which was reduced to 12 hours a day for the next 3 days. Ultrasound, performed after suspecting a complication of prolonged mechanical ventilation, revealed diaphragm weakness.

**Figure 1.**
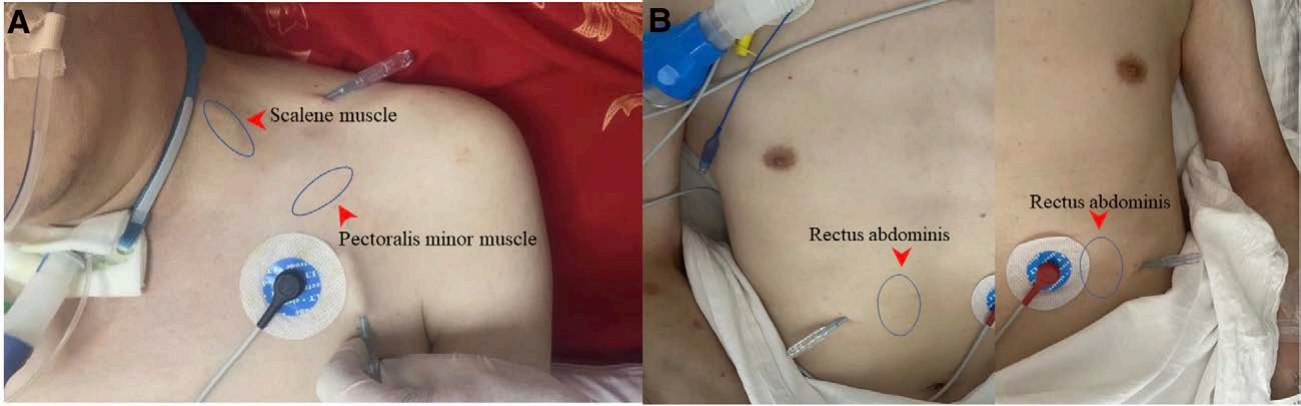
(A) Locations of myofascial trigger point (MTrPs) confirmed in the neck and the chest muscles. (B) Locations of MTrPs confirmed in abdomen area muscles.

**Figure 2.**
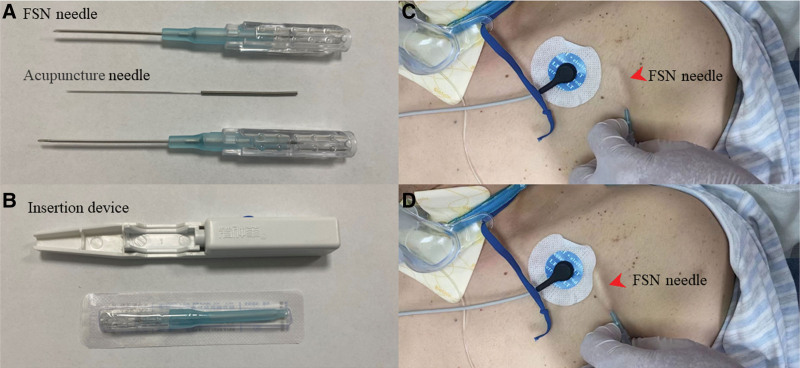
(A) Fu’s subcutaneous needling (FSN) needle and acupuncture needle (0.25 mm diameter, 40 mm length). The steel needle was pulled back 3 mm to select the protrusion of the cannula handle clockwise and to fix it in a slot. (B) FSN needle and insertion device. (C and D) The swaying movement in the subcutaneous layer above the chest muscles.

**Figure 3.**
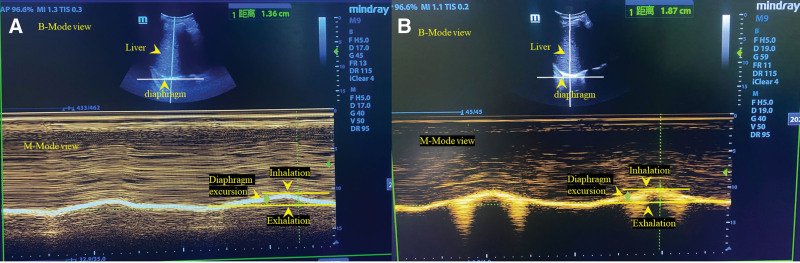
(A) Diaphragmatic excursion (1.36 cm) was conducted prior to the second treatment. (B) Diaphragmatic excursion (1.87 cm) was conducted after the last treatment.

To improve the patient condition and facilitate early weaning from the ventilator, we employed FSN therapy.^[8,11]^ Results from touch-based physical examination revealed the presence of myofascial trigger points (MTrPs) around his neck, chest, and abdomen (Fig. 1A and B). Therefore, he was subjected to FSN therapy, which entailed the insertion of the needles into the subcutaneous layer using an insertion device, followed by 100 horizontal swaying movements from side to side in 1 minute (Fig. 2C and D). The patient received 11 FSN sessions over a 3-week period. To assess his diaphragmatic function, we conducted a total of 3 ultrasound assessments, with the first assessment conducted before the second treatment, a second assessment after the second treatment, and the third assessment after the final treatment (as shown in Fig. 3A and B).

## 3. Outcomes

**Figure 4.**
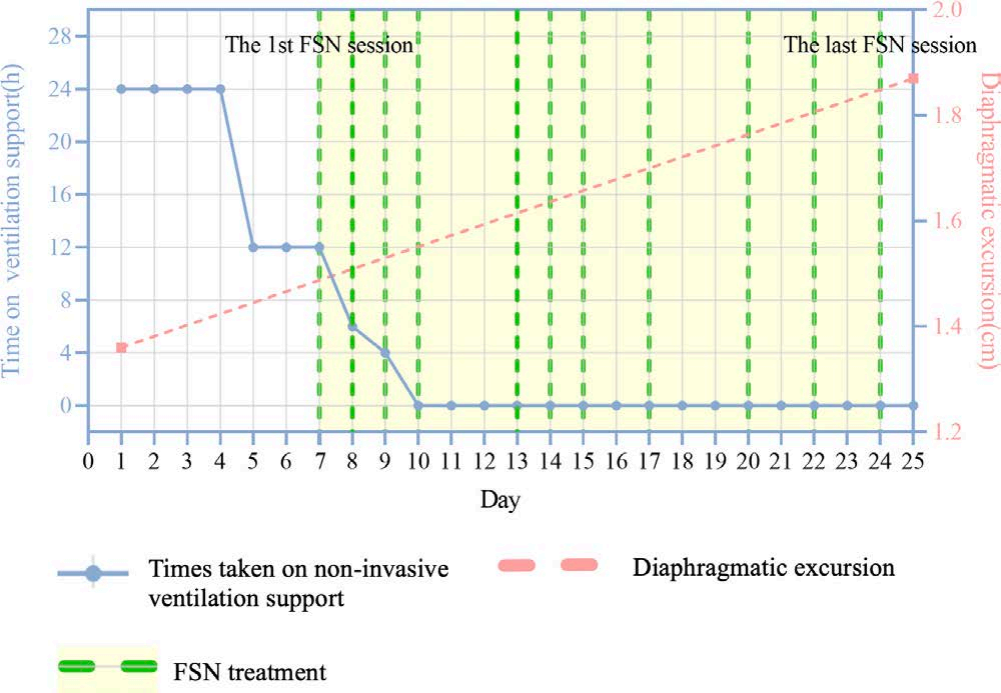
Timeline of daily time on noninvasive ventilation support.

Under the premise that ventilator parameters and ventilation mode did not change, significantly decreased daily time on ventilation was shown after FSN treatment. On day 2 of FSN treatment, ventilation support was needed for about 4 hours at night. On day 3, the patient was successfully weaned from the ventilator, and symptoms such as dyspnea or tachypnea improved as well (20 breaths per minute). Besides, we observed increases both in diaphragmatic excursion (DE) and thickening fraction of the diaphragm (TFdi) after the second treatment and after the last treatment. Just after the second treatment, the DE increased from 1.36 cm to 1.52 cm immediately, and after the last treatment, the DE was 1.87 cm (Fig. 4). Similar increases were found in the TFdi, from 26.3% to 31.2%, and up to 33.3% after the last treatment. The assessment of diaphragmatic function was performed during unassisted breathing, and the potential underestimate of diaphragmatic function could be excluded. Throughout the course of FSN treatment, no obvious adverse events and vital signs affected were observed. In addition, as of now, there has been a 12-month follow-up period and the patient has not relapsed (Fig. 4).

## 4. Discussion

To the best of our knowledge, this is the first report describing efficacy of FSN as an adjunctive therapeutic approach for the management of diaphragmatic dysfunction. Notably, the patient was successfully extubated after 3 FSN treatments, and significant improvement was observed in DE and TFdi.

The patient in the present case study was subjected to FSN therapy, a method that combines traditional Chinese acupuncture and dry needling. Studies have reported that FSN is efficacious against various soft tissue injuries and medical issues,^[7–9,11,12]^ recent studies^[13,14]^ reveal that stretching subcutaneously can influence adjacent VA and FSN may enhance gastrointestinal motility and decrease gastric drainage volume. Unlike traditional Chinese acupuncture methods that may involve deep needle insertion, FSN entails the shallow insertion of a disposable needle into the subcutaneous area near the affected muscle, referred to as the “tightened muscle” with one or more MTrPs.^[8]^ The needle is then manipulated using a horizontal swaying movement, rather than the deeper stimulation techniques used in traditional acupuncture.^[10]^

The current therapeutic options for the management of diaphragmatic dysfunction are limited; conventional treatment strategies that target only 1 mechanism ineffective. Although early ICU rehabilitation programs have benefits, they are limited by various factors key among them patients complex conditions, lack of awareness among staff, and low staffing. These challenges significantly hinder rehabilitation outcomes across the ICU setting.^[15]^ Therefore, the development and implementation of a comprehensive treatment plan for ICU survivors throughout their rehabilitation journey are imperative to effective management, and clinicians have recommended inspiratory muscle training for patients with weak inspiratory muscles or trouble weaning from ventilation.^[16]^ In this case study, however, a major barrier to effective inspiratory muscle rehabilitation is that the patient must have adequate cognitive status and actively participate in the exercise. Thus, inspiratory muscle training could not be applied to the patient in the present case.

The efficacy of dry needling and acupuncture as adjunctive treatment therapies for respiratory diseases, including chronic obstructive pulmonary disease and asthma, has been extensively studied. Results indicate that these treatments are not only well-tolerated but also provide additional benefits beyond routine care. Acupuncture could improve the symptoms of a patient with severe COVID-19, including breath rate, SpO2, and the heart rate.^[17]^ Results from a recent randomized pragmatic trial revealed that the addition of acupuncture treatment to routine care was associated with improved disease-specific and health-related quality of life in patients with allergic asthma.^[18]^ Another study demonstrated that a single session of dry needling, applied over active MTrPs in the scalene muscles, effectively improved inspiratory vital capacity in individuals with mechanical neck pain.^[19]^ Consistently, results from another study revealed that the addition of dry needling over regions related to respiration to usual treatment was associated with a reduction in symptoms and an increase in exercise capacity in chronic obstructive pulmonary disease patients of medium severity.^[20]^ Collectively, these findings suggest that acupuncture and dry needling have the potential as adjunctive treatments for the management of respiratory diseases.

FSN therapy combines the advantages of traditional acupuncture and dry needling and is particularly effective in reducing muscle stiffness. Studies have shown that FSN therapy can suppress musculoskeletal pain by not only eliminating MTrPs but also reducing muscle tissue hardness.^[8]^ Similarly, the dry needling technique also has the ability to reduce muscle stiffness^[21]^ and improve various parameters of neuromuscular function, such as tone, relaxation, and creep.^[22]^ Recently, FSN therapy was found to improve the morphological structure and function of mitochondria within tightened muscles, as evidenced by the upregulation of mitochondrial citrate synthase and Complex II as well as elevated muscle energy metabolism.^[23]^

In the present case, FSN treatment aims to restore and enhance the function of respiratory muscles by eliminating MTrPs and alleviating muscle tightness and cramps.^[10,24]^ Abdominal muscles play a crucial role in respiration and contribute to effective coughing and secretion clearance during forceful expiratory efforts.^[25]^ Results from a recent study revealed that patients with impaired abdominal muscle function are at risk of failing to be liberated from mechanical ventilation.^[26]^ Another study demonstrated that the application of functional electrical stimulation over the abdominal muscles could improve cough in people with spinal cord injury.^[27]^ Fascia, which connects the rectus abdominis and intercostal muscle to the diaphragm, transfers the effects of FSN to the painful area. Studies have also shown that inactivating MTrPs in the abdomen and chest muscles can result in the disappearance of associated MTrPs in the diaphragm.^[28]^

The patient received multiple other rehabilitation therapies, including neuromuscular electrical stimulation, manual hyperinflation, and thorax vibration, in accordance with the current standard of primary care practice. It is important to note that the magnitude of improvement observed in this case report may be influenced by the background of usual care received by the patient. Despite these limitations, the results of this case report are encouraging. Within the context of integrative therapies explored in this paper, FSN was found to supplement rather than supplant the impact of usual care. During the treatment course, however, he was not administered drugs with potential favorable effects on respiratory muscle function, such as theophylline or antioxidants. Collectively, these results suggest that FSN is an efficacious and safe adjunctive therapy for the treatment of diaphragmatic dysfunction in critically ill patients. However, this efficacy needs to be validated by further studies.

## 5. Conclusion

Our study suggests that FSN, a type of dry needling, is efficacious for early active rehabilitation of patients with diaphragmatic dysfunction and severe neurologic disabilities. Despite being limited to a single case report, it is clear that the ease of administration and feasibility of FSN makes it a promising addition to the repertoire of respiratory rehabilitation strategies, especially considering the unsatisfactory outcomes observed with conventional treatments. In conclusion, FSN is a promising therapeutic strategy for improving recovery outcomes in critically ill patients with diaphragmatic dysfunction-associated weaning difficulties, but further research is still required to establish the effects of FSN on diaphragmatic function.

## Author contributions

**Conceptualization:** Hu Li.

**Investigation:** Tianyu Bai.

**Methodology:** Cong Cong Yang.

**Project administration:** Hu Li, Tianyu Bai.

**Supervision:** Jian Sun, Zhonghua Fu.

**Validation:** Hu Li, Tianyu Bai.

**Visualization:** Hu Li, Tianyu Bai.

**Writing – original draft:** Hu Li.

**Writing – review & editing:** Zhonghua Fu, Li-Wei Chou.
